# Mass and Stiffness Deconvolution in Nanomechanical Resonators for Precise Mass Measurement and In Vivo Biosensing

**DOI:** 10.1021/acsnano.4c03391

**Published:** 2024-07-29

**Authors:** Gourav Bhattacharya, Stuart McMichael, Indrianita Lionadi, Pardis Biglarbeigi, Dewar Finlay, Pilar Fernandez-Ibanez, Amir Farokh Payam

**Affiliations:** † Nanotechnology and Integrated Bioengineering Centre, School of Engineering, Ulster University, Belfast BT15 1AP, U.K.; ‡ Department of Pharmacology & Therapeutics, Whelan Building, University of Liverpool, Liverpool L69 3GE England, U.K.

**Keywords:** nanomechanical resonator, mass measurements, stiffness, lipids, biomolecules

## Abstract

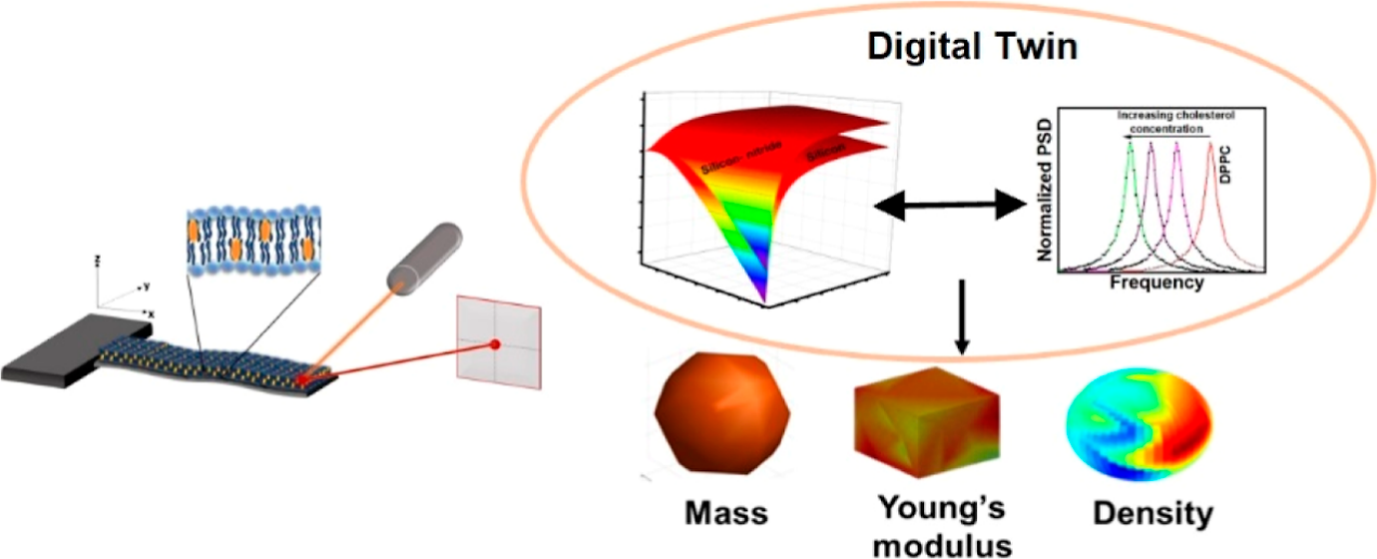

Nanomechanical sensors, due to their small size and high sensitivity to the environment, hold significant promise for various sensing applications. These sensors enable rapid, highly sensitive, and selective detection of biological and biochemical entities as well as mass spectrometry by utilizing the frequency shift of nanomechanical resonators. Nanomechanical systems have been employed to measure the mass of cells and biomolecules and study the fundamentals of surface science such as phase transitions and diffusion. Here, we develop a methodology using both experimental measurements and numerical simulations to explore the characteristics of nanomechanical resonators when the detection entities are absorbed on the cantilever surface and quantify the mass, density, and Young’s modulus of adsorbed entities. Moreover, based on this proposed concept, we present an experimental method for measuring the mass of molecules and living biological entities in their physiological environment. This approach could find applications in predicting the behavior of bionanoelectromechanical resonators functionalized with biological capture molecules, as well as in label-free, nonfunctionalized micro/nanoscale biosensing and mass spectrometry of living bioentities.

## Introduction

Recent advances in micro- and nanofabrication technologies have led to the development of increasingly smaller mechanical transducers.^1−4^ These devices are capable of detecting forces, masses, mechanical properties, and motions involved in biomolecular interactions and study the principles of basic phenomena in biological processes.^1,2,4−10^ Hence, over the past decade, mass measurements with nanomechanical devices have systematically improved, reaching a point where they offer an intriguing capability for an innovative approach to mass spectrometry.^2,11−15^ Nanoelectromechanical system (NEMS) resonators, in particular, exhibit extreme sensitivity to the added mass of adsorbed particles.^3,15−18^ This sensitivity has led to significant advances, including the detection of the mass of individual proteins,^19^ nanoparticles,^14,20^ and large biomolecules.^10,21^ Moreover, there have been demonstrations of near-atomic-scale mass resolution,^13,22^ and even the probing of the mass and motion of bacteria and their reaction to antibiotics.^23−28^ These advancements have set the stage for the emergence of nanoelectromechanical system-based mass spectrometry (NEMS-MS) as a crucial analytical tool in proteomics, structural biology, and nanoparticle detection.^9,19,29^

However, accurate mass quantification faces challenges when the mechanical characteristics of nanoresonators are affected by changes in the mass and size of the adsorbent.^30,31^ This interference can compromise the precision of mass detection, leading to potential discrepancies in the detected masses.^30^ Additionally, the size-specific modification of diffusion and attachment kinetics of biomolecules on the nanomechanical surface results in anomalous frequency shifts during the measurement of resonator frequencies.^31^

In prior studies, the influence of the changing of the spring constant and the mass of the adsorbed layer on resonators was demonstrated.^31−34^ It was elucidated that changes in the stiffness of the resonators corresponded to stress alterations, particularly on one side of the resonators, in this case, the cantilever.^31^ However, in these studies, the impact of the mass of the adsorbed layer was disregarded, primarily due to the significantly greater mass of the cantilever compared to that of the adsorbed layer.

While certain studies have made efforts to examine the influence of the mass and spatial distribution of particles on nanoresonators,^35^ the origin of observed anomalous frequency shifts remains unclear. Furthermore, the critical size and properties of the adsorbed layer, leading to the change in the polarity of frequency shifts, have yet to be conclusively determined.

Moreover, the alteration of mechanical characteristics and the frequency response of microcantilevers make use of standard equations for mass spectrometry^30,33^ prone to significant errors. Additionally, since most biological entities exist in their physiological environments, detecting their mass requires a fluid environment, which further affects the mechanical characteristics and sensitivity of microcantilevers.

**Figure 1:**
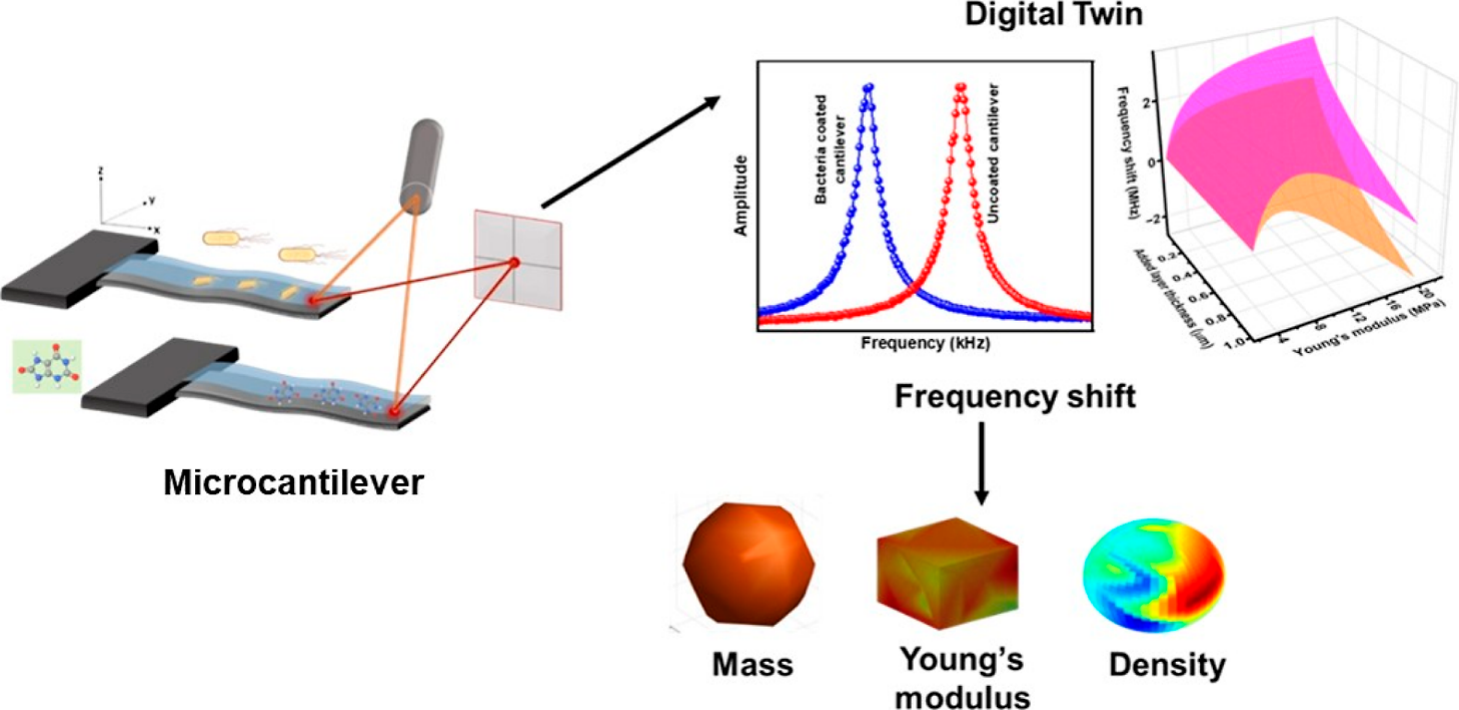
Schematic of microcantilevers for biosensing, mass detection, and characterization.

In this work, employing the digital twin concept, we have developed an integrated theoretical–experimental methodology to elucidate the layer adsorption mechanism and the characteristics of nanomechanical resonators (schematic in Figure 1). Our approach aims to explore the origin of observed anomalous frequency shifts and enhance the mass spectrometry of nanoelectromechanical sensors under ambient conditions.

Theoretically, we have formulated a model to determine the direction of frequency shifts associated with the critical size and properties of the adsorbed layer. We present an equation that considers the interplay between stiffness and mass changes to convert the measured microcantilever resonance frequency into the biomolecule and adsorbed layer mass. Building on this theoretical foundation, we present an experimental approach to measure the mass and mechanical properties of protein and lipid bilayers with different cholesterol concentrations as well as the mass of molecules and living bacteria without using fluidic systems in an ambient environment.

## Results and Discussion

### Theoretical Modeling

The standard operating principle of microcantilever-based mass sensing hinges on detecting a deviation in the natural resonance frequency Δ*f* of the microcantilever upon attachment of an analyte. This alteration in the cantilever’s resonance frequency is then quantified as the mass Δ*m* of the attached analyte, determined by the following equation^30^
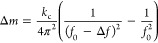
1where *k*_c_ is the spring constant of the microcantilever, *f*_0_ is the natural resonance frequency of the microcantilever, and Δ*f* is the change in the frequency.

However, as mentioned above, when the analyte is attached to the cantilever, the stiffness of the cantilever is changed due to the analyte properties, geometry, and size. The spring constant considering the effect of the analyte *k*_c+a_ can be described by
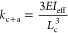
2where *L*_c_ is the length of the cantilever and *EI*_eff_ is modeled by^36−38^
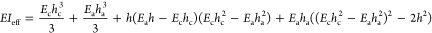
3where *E*_c_ and *E*_a_ are the Young’s modulus of the cantilever and the added layer, *h*_c_ and *h*_a_ are the thicknesses of the cantilever and the added layer, respectively, and *h* is the position of the neutral axis because of the added top layer thickness
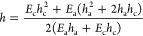
4

The density of the cantilever and the added layer is denoted by ρ_c_ and ρ_a_, respectively.

A change in the spring constant of the cantilever can lead to a positive frequency shift, which in turn leads to the inaccuracy in eq 1. So, considering the changes in the spring constant of the cantilever due to the added layer, we can decouple the effect of the stiffness variation from the added mass and use the following equation to measure the added mass of the adsorbed layer
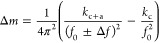
5

The Young’s modulus of the added layer can also be quantified by considering eqs 2–4 and measured spring constant.

Furthermore, from the above equations and the frequency of the cantilever (see the Supporting Information), we can determine the critical thickness of the adsorbed layer, which will cause the resonant frequency to increase (see the Supporting Information)
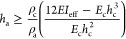
6

A similar approach can be used to determine the critical density and Young’s modulus of the adsorbed layer (see the Supporting Information).

### Numerical Analysis

Initially, our presented model is utilized to examine the influence of varying thicknesses of coated biomaterials and their physical characteristics on the frequency response of microcantilevers, which is calculated by the following equation
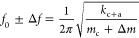
7

In the case of the pristine cantilever with no additional mass (Δ*f* = 0), eq 7 can be converted to the normal equation of the microcantilever where Δ*m* = 0 and *k*_c+a_ = *k*_c_.

**Figure 2:**
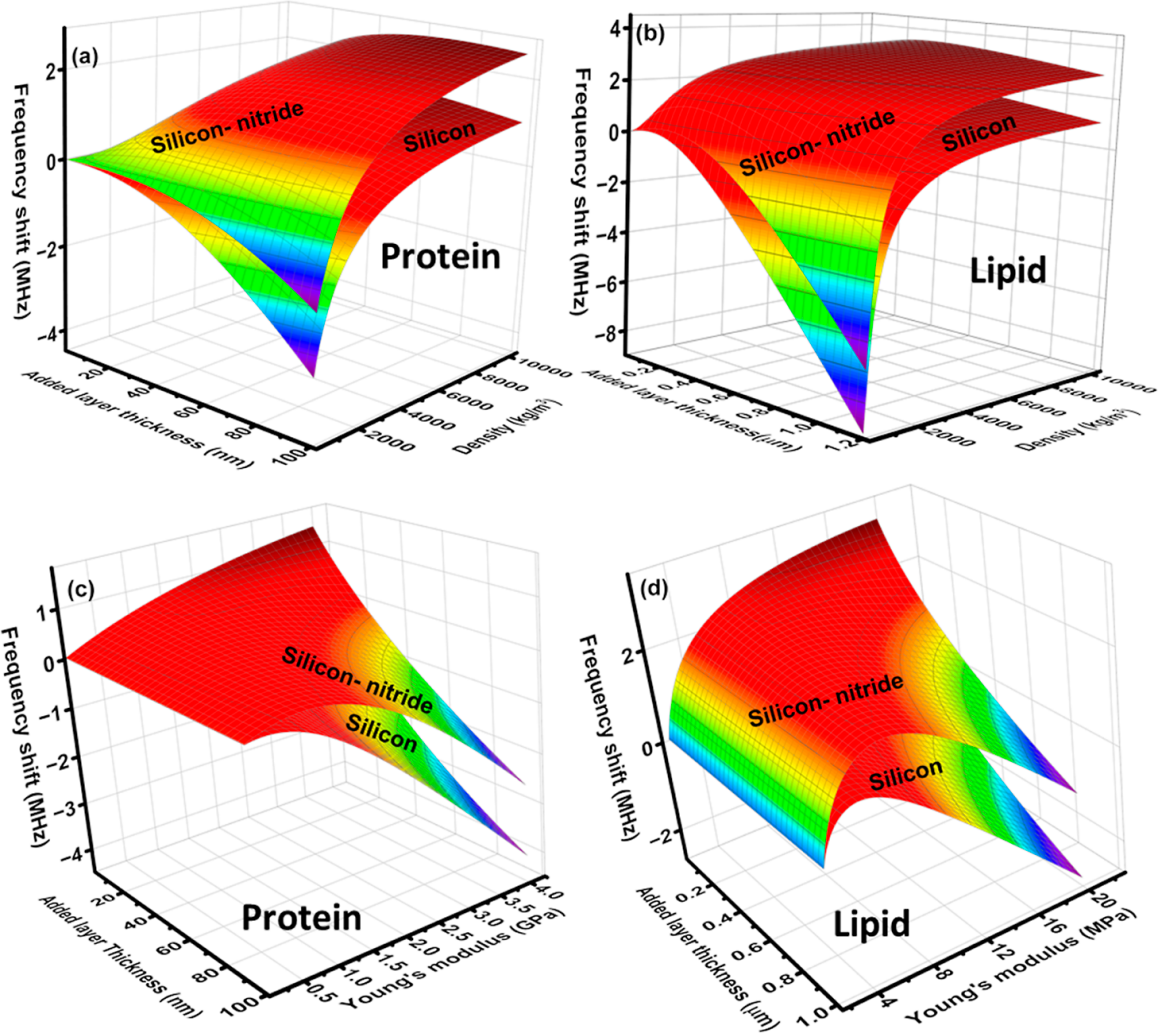
Numerical simulation results of frequency shift due to the changes of thickness and density of coated (a) protein and (b) lipid layers and frequency shift because of the changes of thickness and Young’s modulus of coated (c) protein and (d) lipid bilayers on silicon and silicon nitride microcantilever surfaces.

Specifically, we investigated silicon and silicon nitride cantilevers, each sharing identical dimensions (length: 3 μm, width: 1.5 μm, and thickness: 25 nm), with the addition of a protein layer of variable thicknesses. In the first series of simulations, we computed alterations in the natural frequency of the modified cantilever in response to changes in the thickness of the protein layer and its density. The resulting variations are depicted in Figure 2a. Notably, we identify a critical frequency, thickness, and density, whereupon the polarity of the frequency shift reverses. It is observed that the positive frequency shift for the silicon nitride cantilever surpasses that of the silicon cantilever; conversely, a more pronounced negative shift was evident in the case of the silicon-based cantilever. This observation suggests that the frequency shift attributed to the coated biomaterial is contingent not only upon its inherent properties but also on those of the pristine cantilever. To assess the universality of our mathematical model, we investigated another biomaterial, namely, a lipid bilayer composed of dipalmitoylphosphatidylcholine (DPPC) and cholesterol. The alteration in the natural frequency of the modified cantilever was plotted against layer thickness and density, as shown in Figure 2b. Interestingly, the observed variation closely resembled that of the protein-coated cantilever; however, the magnitude of the positive frequency shift was notably greater than that observed with the protein coating, which can be explained by the higher Young’s modulus of the protein than the lipid bilayer.

Subsequently, we examined the impact of added layer thickness and Young’s modulus on the frequency response of the modified cantilever for both protein and lipid coatings, as depicted in Figure 2c,d, respectively. The observed variations paralleled those observed when the densities of the added layer were determined. Furthermore, we noted a reversal of polarity in both cases, defining the Young’s modulus at which this occurs as the critical Young’s modulus. It is important to highlight that to determine the critical density and critical Young’s modulus for the chosen biomaterials, appropriate ranges were selected. For both protein and lipid coatings, the density range was chosen as 100–10,000 kg/m^3^, while Young’s modulus range was set as 0.1–4 GPa for protein and 0.1–20 MPa for the lipid bilayer.

**Figure 3:**
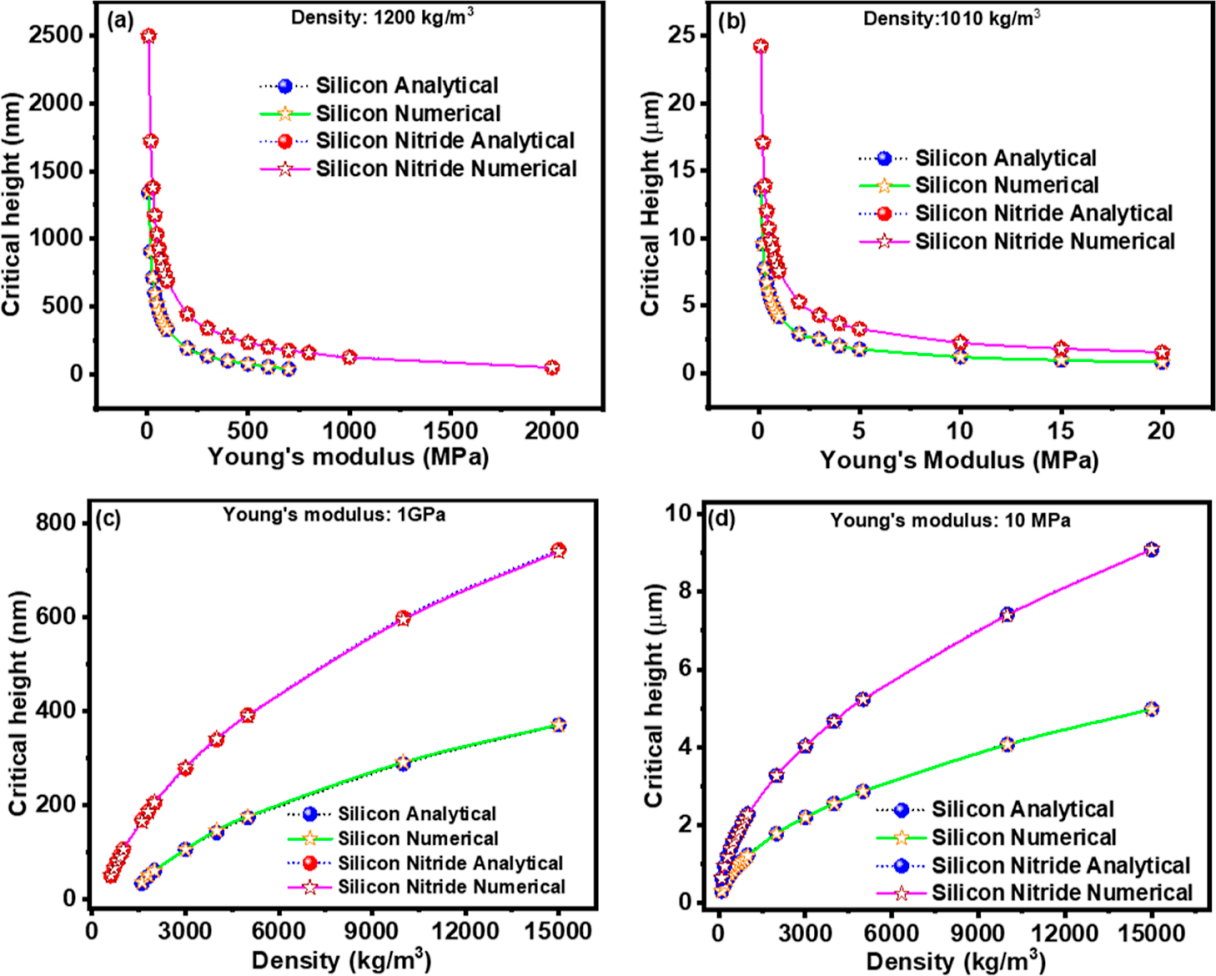
Comparison between the critical height as a function of variable Young’s modulus obtained from numerical simulations and developed analytical equations for (a) protein layer and (b) lipid bilayer for silicon and silicon nitride cantilevers. Comparison between the critical height as a function of variable density obtained from numerical simulations and developed analytical equations for the (c) protein layer and (d) lipid bilayer for silicon and silicon nitride cantilevers.

In the subsequent phase, we verify the accuracy of our derived analytical equations for determining these crucial values by comparing them with the corresponding results from numerical simulations. For this comparative assessment, we utilize cantilevers analogous to those employed in the preceding numerical simulations. Figure 3a–d depicts the variation of critical height across ranges of Young’s modulus, as well as the variation of critical height relative to different densities, for both silicon and silicon nitride cantilevers.

The findings reveal significant alignment between numerical simulations and our derived analytical equations. Through this analysis, it is observed that the critical height diminishes with increasing Young’s modulus. Specifically, in the case of protein coating (Figure 3a), the silicon nitride cantilever exhibits a higher critical height compared to the silicon cantilever. Additionally, for the silicon-based cantilever, the halt of frequency polarity reversal occurs at a relatively lower Young’s modulus compared to the silicon nitride-based cantilever. A similar trend is observed in the case of lipid bilayer-coated cantilevers (Figure 3b), albeit with a substantially higher critical height (approximately 10-fold) compared to protein-coated counterparts. Furthermore, unlike the protein-coated cantilever, the lipid-coated silicon-based cantilever demonstrates frequency reversal across the entire Young’s modulus range. When critical height is examined as a function of varying densities, a nonlinear increase in critical height with increasing density of the added layer is noted for protein-coated cantilevers (Figure 3c). Consistently, the silicon nitride-based cantilever achieves a higher critical height compared to the silicon-based cantilever, mirroring the trends observed in the critical height versus Young’s modulus plots. A similar pattern is observed for lipid bilayer-coated cantilevers (Figure 3d), with a substantially higher critical height (approximately 10-fold) attained.

**Figure 4:**
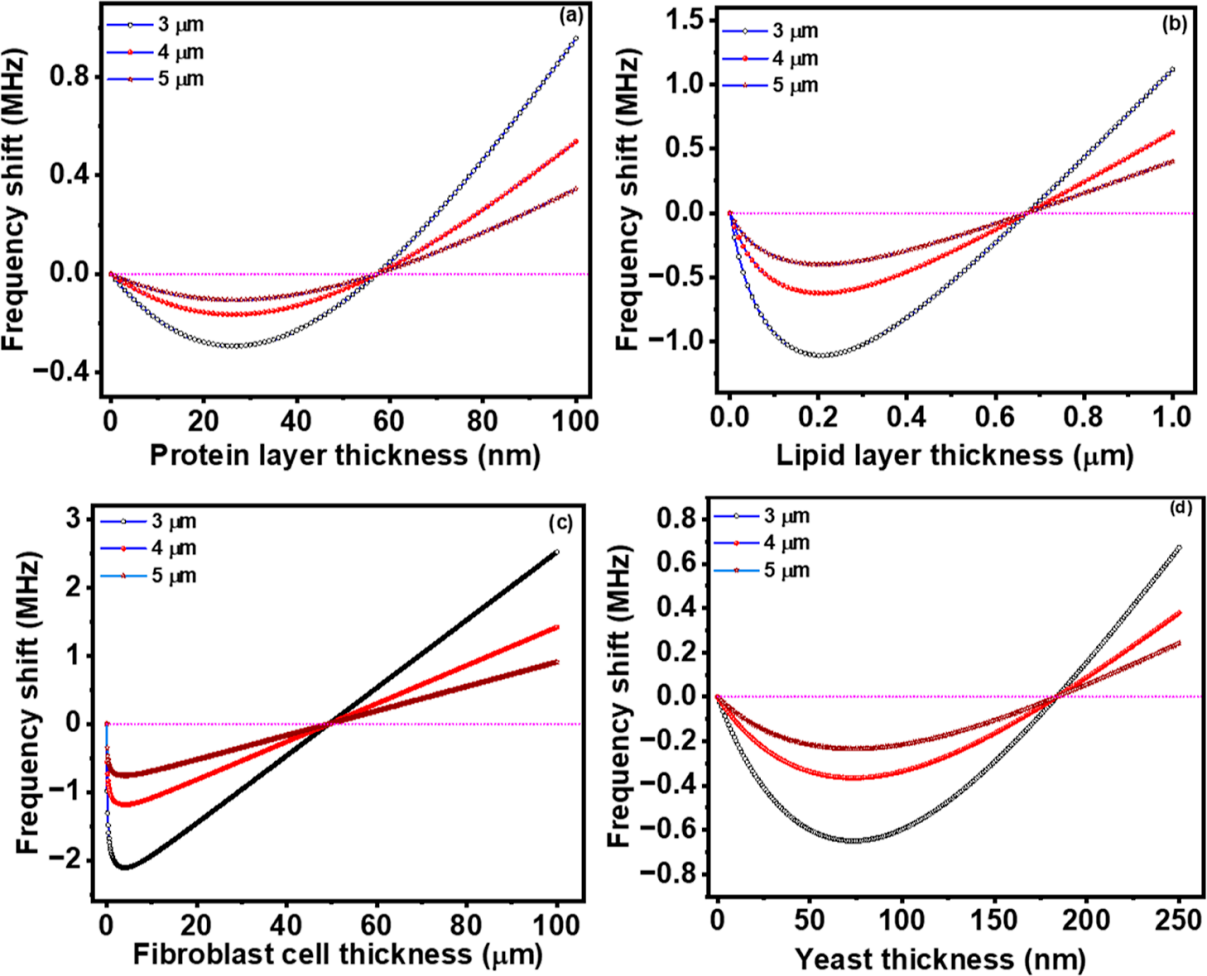
Variation of resonant frequency shift as a function of (a) protein layer thickness, (b) lipid layer thickness, (c) fibroblast thickness, and (d) yeast thickness for different cantilever beam lengths.

Subsequently, we extend our analysis to consider different biomaterial coatings on our pristine cantilever (protein, lipid, fibroblast cell, and yeast; the physical properties of all these biomaterials are provided in Table 1 ). The frequency shift as a function of added biomaterial thickness is measured and is plotted in Figure 4. For these computations, the density and Young’s modulus of the added layers are held constant (as obtained from the literature). Throughout the calculations, the width and thickness of the cantilevers remain fixed (width: 1.5 μm and thickness: 25 nm), while the length of the pristine cantilever varies.

**Table 1:** Young’s Modulus and Density of 4 Different Biomaterials

biomaterials	Young’s modulus	density (kg/m^3^)
protein	1 GPa	1220
phospholipid (+cholesterol)	10 MPa	1010
fibroblast cells	2 kPa	1050
yeast	127 MPa	1100

The graph in Figure 4a illustrates the variation of the frequency shift concerning protein layer thickness. Initially, as the protein thickness increases, the frequency of the modified cantilever decreases. Subsequently, with further increases in layer thickness, the frequency of the modified cantilever gradually transitions toward less negative values. At a certain thickness, the frequency shift reaches zero, and beyond this point, an increase in thickness results in a positive polarity of the frequency shift. The thickness of the added layer at which the polarity of the frequency shift reverses termed the critical height was measured and determined to be 58 nm. Notably, this critical height was found to be independent of the cantilever length. Similarly, in the case of the lipid bilayer (Figure 4b), the critical height was measured to be 680 nm. For fibroblast cells (Figure 4c), the critical height was determined to be 49.4 μm, while for yeast (Figure 4d), it was found to be 184 nm. Considering the values of density and Young’s modulus of protein, lipid, fibroblast cell, and yeast, we can observe that the critical height has a linear relationship with their Young’s modulus. As their density is close to each other and there is a significant difference in their Young’s modulus, for the protein which possesses a higher Young’s modulus, the critical height is lowest, while the critical height for cells with very low Young’s modulus is very high to cancel the effect of low Young’s modulus to increase the changes of stiffness dominance than mass when the cells are adsorbed on cantilever surface.

### Experimental Analysis

**Figure 5:**
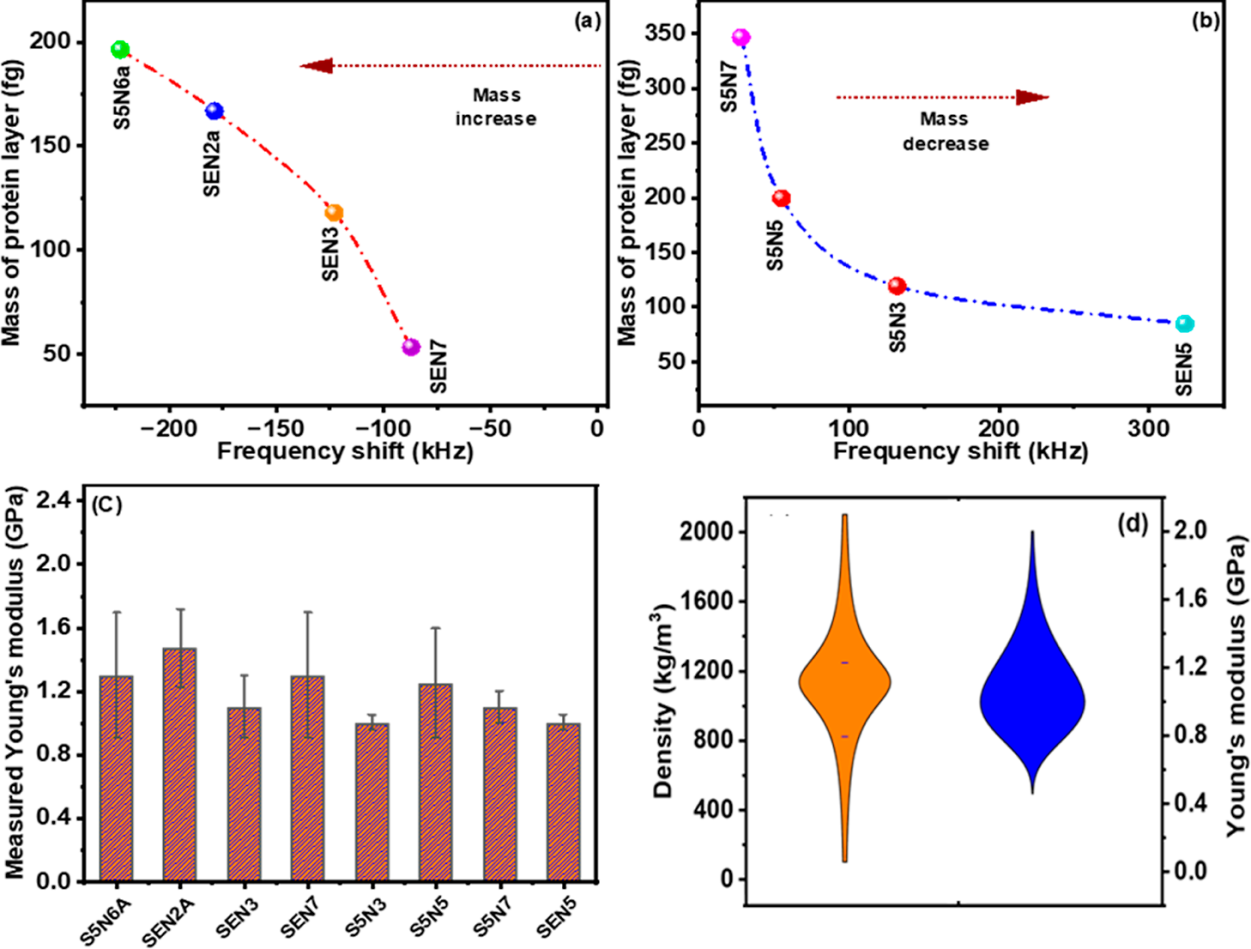
Calculation of mass of the protein layers using our mathematical equation for different cantilevers (a) from the negative frequency shift and (b) from the positive frequency shift. (c) Calculated Young’s modulus of the protein layer for the 8 cantilevers. (d) Measurement of the density and Young’s modulus of the protein layer deposited on the surface of different cantilevers.

In the subsequent phase of our investigation, our objective was to assess the mass and other physical attributes of the introduced biomaterials using our proprietary model and to compare our model’s outcomes with mathematical equations derived from the existing literature. To validate our mathematical formulation, we selected a series of cantilevers documented in previous experimental studies.^31^ These cantilevers were subjected to a biotinylated bovine serum albumin and streptavidin protein coating, and the resulting frequency shift due to protein layer deposition, along with the modified spring constant of the cantilevers, was documented. The dimensional properties, frequency, spring constant of the cantilevers, and the corresponding frequency shift and modified spring constant as a function of the added protein layer are detailed in Table 2. Notably, some cantilevers exhibited a negative shift, while others displayed a positive frequency shift. Utilizing eq 5 and considering the spring constant of both the pristine and modified cantilevers, we determined the mass of the added layer and depicted the mass of the cantilever as a function of the frequency shift for different cantilevers (Figure 5a,b). Our approach incorporated the stiffness of the modified cantilever to decouple its effect in computing the mass of the added layer. In contrast, an alternative model described in the literature computes the layer’s mass solely based on the pristine cantilever’s stiffness (eq 1). The mass of the added layer was also estimated using this model and plotted in Supporting Information Figure S1a,b. Notably, the mass calculated from the negative shift followed a consistent trend for both models, with an observed increase in mass with increasing frequency shift. Conversely, it is widely acknowledged that for positive frequency shifts, the stiffness effect outweighs that of the mass effect. Therefore, it was anticipated that for higher positive frequency shifts, the effective mass of the added layer would decrease. With our mathematical model, which accounted for the stiffness of the added layer, we successfully predicted a similar trend (Figure 5b). However, the mathematical model from the literature,^31^ which neglected to decouple the stiffness of the added layer, failed to anticipate this phenomenon.

**Table 2:** Different Properties of Cantilevers Chosen to Calculate the Mass and Young’s Modulus

cantilevers	pristine frequency (MHz)	pristine spring constant (N/m)	modified frequency (MHz)	modified spring constant (N/m)	length (μm)	width (μm)	height (nm)
S5N6a	2.93	0.0227	2.707	0.033	3.1	1.4	25
SEN2a	2.237	0.011	2.058	0.016	2.8	1.6	25
SEN3	2.246	0.0125	2.123	0.0162	2.9	1.6	25
SEN7	2.439	0.0142	2.352	0.016	2.8	1.8	25
S5N3	1.264	0.0091	1.396	0.0133	5	1.5	25
S5N5	1.767	0.0131	1.822	0.0202	3.3	1.4	25
S5N7	1.3	0.0072	1.328	0.0133	4.7	1.3	25
SEN5	2.363	0.0201	2.687	0.0202	2.8	1.6	25

Our mathematical model not only facilitates the determination of the mass of the added layer but also offers the capability to predict the Young’s modulus and density of the said layer (eqs 2–7 and Supporting Information). Through our approach, we have effectively disentangled the influence of mass and Young’s modulus. The Young’s modulus of the protein layers deposited on the previously mentioned eight cantilevers was measured and is depicted in Figure 5c. The average Young’s modulus across all cantilevers was found to fall within the range of 0.8–1.6 GPa, closely aligning with values reported in the literature (approximately 1 GPa).^31^ Subsequently, we examined several additional cantilevers featuring protein deposition on their surfaces and calculated the average thickness and density of the added protein layers (Figure 5d). The resulting average Young’s modulus was approximately 1.1 GPa (within the range 0.7–1.8 GPa), while the average density was measured to be approximately 1200 kg/m^3^, both of which closely correspond to values reported in the existing literature. It is noteworthy to mention that a single Young’s modulus for any viscoelastic biomaterial may lack precision, and our experimental measurements of Young’s modulus for the protein layer revealed a range of values rather than a singular one, as depicted in Figure 5d.

To extend our analysis to probe the mechanical properties of a lipid bilayer as an adsorbate layer, we employed a zwitterionic synthetic lipid, 1,2-dipalmitoylphosphatidylcholine (DPPC), to formulate a lipid bilayer, which was then deposited onto the pristine cantilever. Subsequently, employing our methodology, we disentangled the mass and Young’s modulus, thereby concurrently measuring both parameters for the added DPPC layer.

In the subsequent phase, we introduced varying mole percentages (mol %) of cholesterol molecules into the DPPC mixture, ranging from 10 to 50 mol %.

Initially, we measured the frequency of the cantilevers and the spring constants as a consequence of the deposition of adsorbate lipid and lipid/cholesterol layers. In our measurement, we also considered a maximum 2% error in the dimensions of the pristine cantilever, repeated the measurement numerous times, and collected a range of frequency and spring constant data. A representative frequency shift and changes in the spring constant for bilayer-coated and bilayer/cholesterol-coated cantilevers are represented in Supporting Information Figure S2a,b. In this case, the pristine cantilever frequency was measured to be 72.328 ± 0.004 kHz, and the spring constant was calculated as 2.5993 ± 0.12 N/m. From the figures, we observed that initially with the addition of the bilayer and the cholesterol bilayer assembly, the frequency shift was negative, indicating the dominancy of mass; however, at the highest concentration of cholesterol molecules (50 mol %), we observed that the polarity of the frequency shift was reversed, and we obtained a positive frequency shift, confirming the dominating stiffness effect. On the other hand, the variation of the changes in the spring constant at all the concentrations exhibited a gradual increment.

**Figure 6:**
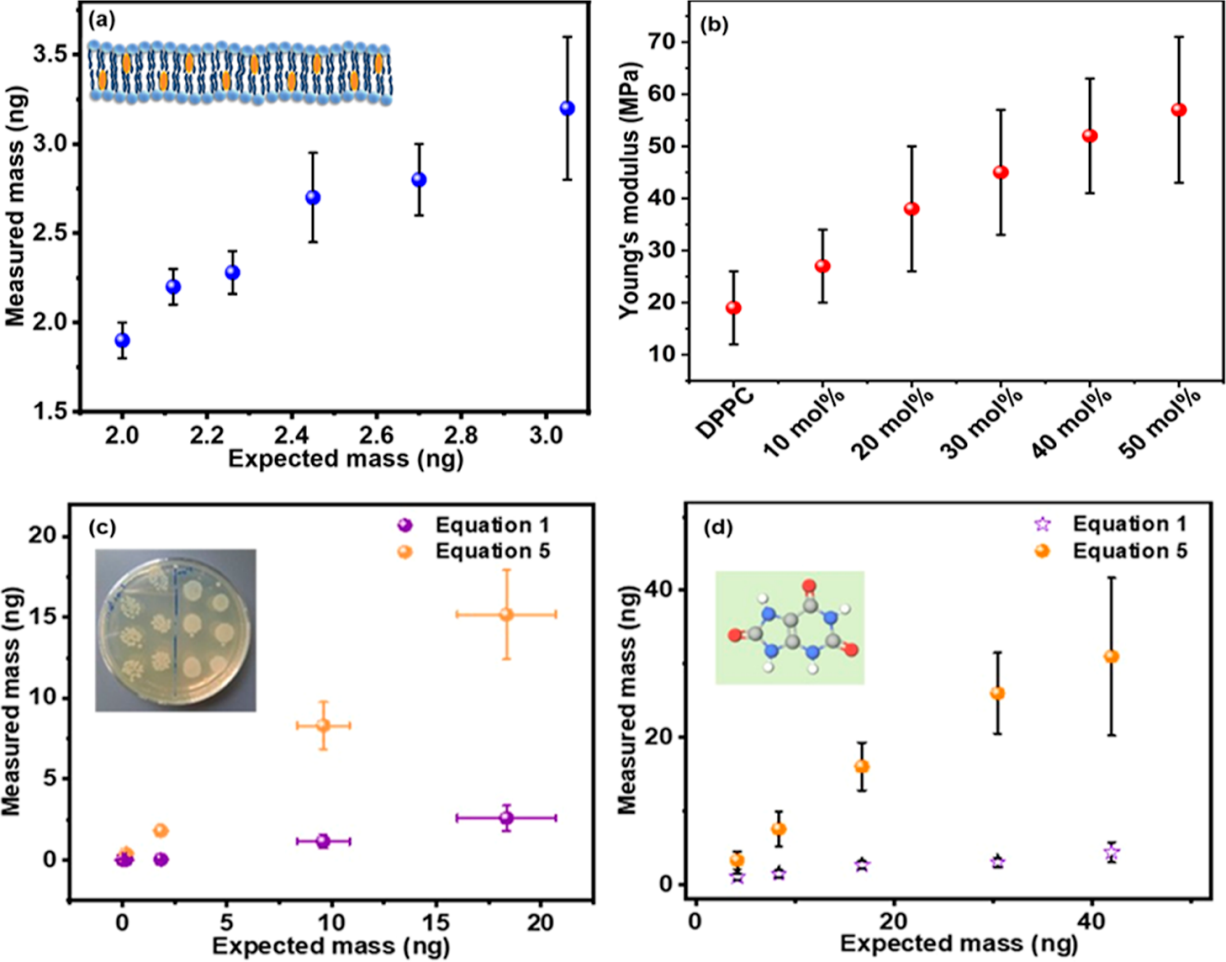
(a) Variations of expected mass (calculated from concentration) and measured mass calculated from eq 5 for DPPC bilayer and DPPC bilayer with different concentrations of cholesterol molecules. (The inset shows the schematic of bilayer/cholesterol assembly.) (b) Variations of calculated Young’s modulus of the DPPC bilayer and DPPC bilayer with increasing mol % of cholesterol molecules. (c) Variation of expected mass (calculated from colony counts) and measured mass calculated from two different mathematical equations for *E. coli* bacteria and (d) variation of expected mass (calculated from concentration) and measured mass calculated from two different mathematical equations for uric acid. (The inset shows the 3d molecular structure of uric acid.)

Then, employing a similar methodology through deconvoluting the mass and spring constant changes of the microcantilever, as we described for the protein layer, we determined the mass of the resulting composite layer and its associated Young’s modulus, and the variation of mass and Young’s modulus for the added lipid bilayer and the lipid bilayer/cholesterol assembly are presented in Figure 6a,b.

From Figure 6a, we found that there is a close agreement between the measured mass of the added DPPC and DPPC/cholesterol composites and their expected masses, calculated from the mol % and concentrations.

Furthermore, we determined the Young’s modulus of the pristine DPPC bilayer to be 19 ± 7 MPa, consistent with reported values in the literature.^39,40^ Additionally, we observed an increase in the Young’s modulus of the composite adsorbate layer with the incorporation of increasing amounts of cholesterol molecules, aligning with the existing literature.^41,42^ For the 10, 20, 30, and 40 mol % of the added cholesterol, the Young’s modulus of 27 ± 7, 38 ± 12, 45 ± 12, and 52 ± 11 MPa was measured, respectively. At the highest concentration of cholesterol molecules (50 mol %), the effective Young’s modulus was calculated to be 57 ± 14 MPa.

In the final segment of our investigation, we executed two experiments to ascertain the mass of (a) living *E. coli* bacteria in physiological conditions and (b) uric acid molecules across various concentrations. *E. coli* bacteria of different concentrations were applied onto an FMV-A cantilever, and subsequently, the frequency shift and spring constant were recorded. To compensate for the added complexity of the fluid environment, we mimic the physiological condition and use live *E. coli* without functionalizing the cantilever surface; in our measurement, we first coated our cantilever with an aqueous phosphate-buffered saline (PBS) solution and measured the change in frequency and spring constant as a consequence of the PBS layer on top of the pristine cantilever (and not directly coated the cantilever with the bacteria). Then, different concentrations of live *E. coli* in the same volume of PBS were prepared and deposited on the pristine cantilever. This approach ensured that the bacterial mass was measured under physiological conditions, ensuring the viability of the bacteria (as the mass of live bacteria differs from that of the dead ones).

While our proposed model can accurately determine Young’s modulus of any adsorbate layer, this capability does not extend to *E. coli* bacteria due to their suspension in a solution (PBS), precluding the formation of an adsorbate layer and thus hindering Young’s modulus estimation. However, there are no such limitations on measuring the mass of introduced bacteria. To measure the mass, we utilized the frequency and spring constant of the PBS-coated cantilever as a reference and then assessed the frequency shift and spring constant when introducing bacteria of various concentrations, thereby accurately determining the mass using our methodology.

The alterations in frequency and spring constant corresponding to different concentrations of *E. coli* (measured in colony-forming units or CFUs) are depicted in Supporting Information Figure S3a. It is apparent from the figure that as the bacterial concentration increases, the negative frequency shift intensifies, while conversely, stiffness augments with escalating CFUs. Subsequently, employing eq 5, we computed the mass of the *E. coli* bacteria, and likewise, we calculated the mass using the method outlined in the existing literature (eq 1). Additionally, the mass of the bacteria was independently calculated based on colony counts. The disparity between the predicted mass (derived from colony counts) and measured mass (determined from mathematical models) is depicted in Figure 6c. Notably, a linear trend is discernible in the mass calculated from our mathematical equation, whereas no such trend is observed in the mass obtained from eq 1. This disparity underscores the superior accuracy of our mathematical model in estimating the mass of the bacteria as in our methodology the changes of stiffness are incorporated, and we decouple the stiffness effect from mass.

Uric acid serves as a vital biomarker, neurotransmitter, and antioxidant, making its detection in body fluids imperative. In the concluding segment of this study, we endeavored to detect varying concentrations of uric acid in the PBS solution. The concentration of uric acid ranged from 5 to 50 μM, and the resultant frequency shift and changes in spring constant were recorded and graphed in Supporting Information Figure S3b. Like the observations with *E. coli*, a more negative frequency shift was noted with increasing uric acid concentration. Furthermore, stiffness demonstrated a direct proportionality to the concentration. Subsequently, the mass of the uric acid molecules was determined by using two distinct mathematical methodologies. The variation between the measured mass and the expected mass (calculated from concentration) is plotted in Figure 6d. Once again, our mathematical model yielded a more accurate estimation of the mass, while the model derived from eq 1 failed to accurately estimate the mass of the uric acid molecules, resulting in an underestimation of the mass.

## Conclusions

In this paper, we proposed a methodology to measure the mass, Young’s modulus, and density of biological entities adsorbed on the cantilever surface. To measure the mass, we deconvolute the effect of stiffness and mass from the measured frequency shift, which leads to significant accuracy in the mass spectrometry. Furthermore, we derived an equation that can calculate the critical height of the added layer which leads to the change of frequency shift direction, and using numerical simulations, we investigate the effect of layer thickness, Young’s modulus, and density on the polarity of frequency shift and validate our analytical equation. Finally, we implemented our methodology to measure the mass and Young’s modulus of a lipid bilayer with different cholesterol concentrations as well as the mass of *E. coli* bacteria in its physiological condition and uric acid and show the high superiority of our methodology in comparison with a standard equation to measure their mass at different concentrations. Our simulations and experimental results show that by using a thinner microcantilever, the sensitivity to measure Young’s modulus of soft materials is increased.

## Methods

### Chemicals

1,2-Dipalmitoylphosphatidylcholine (DPPC) was purchased from Avanti Polar Lipids (USA). Cholesterol molecules of analytical reagent (AR) grade, chloroform, and methanol of HPLC grade were procured from Sigma-Aldrich (Merck, UK).

### Biomaterials for Numerical Simulations

In order to carry out our numerical simulations and analytical model, we have considered coating our pristine cantilever with four biomaterials which are (a) protein, (b) lipid bilayer, (c) fibroblast cell, and (d) yeast. The Young’s modulus and the density of these biomaterials are represented in Table 1.

### Cantilevers

In the first part of the simulation, we utilized silicon- and silicon-nitride-based cantilevers with the following dimensions: length: 3 μm, width: 1.5 μm, and thickness: 25 nm. The density and Young’s modulus for the silicon cantilever were chosen as 2330 kg/m^3^ and 70 GPa, respectively. For the silicon-nitride-based cantilever, the density and Young’s modulus were 3187 kg/m^3^ and 300 GPa, respectively.

The following 8 cantilevers were chosen from the literature:^31^ (1) S5N6a, (2) SEN2a, (3) SEN3, (4) SEN7, (5) S5N3, (6) S5N5, (7) S5N7, and (8) SEN5. They are silicon-based cantilevers. Their respective dimensions along with their spring constants and frequencies are provided in Table 2.

For the measurement of the bacterial mass and the uric acid mass, an FMV-A silicon cantilever (length: 230 μm, width: 30 μm, and thickness: 2.5 μm) was used for the analysis.

### Bacterial Growth

#### Isolation and Identification of Antibiotic-Resistant *E. coli*

Antibiotic resistance (ABR) *E. coli* were isolated from Carrickfergus Wastewater Treatment Works using Chromocult coliform agar (selective agar) and membrane filtration following ISO 9308-1. The ABR was determined using serval antibiotics (ciprofloxacin, tetracycline, ampicillin, ofloxacin, sulfamethoxazole, and trimethoprim) tested to the minimum inhibitory concentration stated by the European Committee on Antimicrobial Susceptibility Testing (EUCAST) and Clinical Laboratory Standards Institute (CLSI). The ABR *E. coli* stock was then stored following the standard protocol for freezing bacteria using glycerol (15%) and cryobeads.

#### Microbially Culture and Analysis

The frozen strains of ABR *E. coli* were used to make a stock plate, using tryptic soy broth (TSB) inoculated with 2 single cryobeads and incubated at 37 °C for 21 h under constant agitation of 120 rpm in an orbital rotary shaker. *E. coli* from the TSB broth were then streak-plated on Chromocult coliform agar and stored at 4 °C in a fridge.

#### Culture and Suspending *E. coli* on PBS

*The E. coli* broth was cultured using 2 colonies from a stock plate and TSB and incubated at 37 °C for ∼21 h under constant agitation of 120 rpm in an orbital rotary shaker. The broth was centrifuged at 4000 rpm for 1 min to form a pellet of bacteria, and the superintend was removed. The bacterial pellet was then resuspended in PBS. The initial concentration was ∼10^8^ CFU/ml, and 10-fold serial dilutions were then performed to have a range of concentrations from 10^7^ to 10^3^ CFU/ml.

#### Enumeration of Microorganisms

Serial dilutions (10-fold) were performed on each sample using PBS. Six drops of 10 μL were plated on tryptic soy agar; this was done for each dilution and incubated at 37 °C for >18 h. The dilution with a countable number of colonies was then enumerated, and the average and associated deviation was calculated for a 5 μL sample which was placed on the cantilever. The reported mass of an *E. coli* cell is 1048 ± 98 fg (∼1 pg); as such, the mass of each sample was estimated by multiplying the number of CFU in a 5 μL sample by 1 pg to get the total mass.

#### Lipid Bilayer Preparation

Lipid bilayer formation was conducted using a protocol reported in the literature.^43^ In summary, we prepared a 2 mM solution of powdered 1,2-dipalmitoylphosphatidylcholine (DPPC), and the first step involved weighing out the appropriate amount of DPPC and adding chloroform/methanol in a ratio of 2:1. Subsequently, the solvents evaporated under a continuous stream of nitrogen for approximately 45 min. To form an aqueous dispersion, water is added to achieve a concentration of 0.3 mg/mL. A milky white dispersion was formed, and this dispersion was then stirred using a magnetic stirrer at 1100 rpm under constant nitrogen flow for 30 min. The solution was then placed at 60 °C for 1 h to facilitate swelling, followed by an additional 30 min stirring step at 1100 rpm at room temperature. Subsequently, the solution was sonicated using a probe sonicator for 1 h at 60 °C until the milky white solution turned completely clear. Additional water was added to dilute the solution and finally drop-cast over the cantilever. The cantilever was then dried at ambient temperature, and a uniform coating was obtained.

The cholesterol molecules were dissolved in chloroform, and the desired mol % of the cholesterol molecules were mixed with the aqueous DPPC solution. A uniform dispersion was prepared using a vortex mixture, and the resultant solution was drop-cast and dried over the cantilever to achieve uniform coatings.

#### Uric Acid Solution

Uric acid and PBS tablets of AR grade were procured from Sigma-Aldrich (UK) and employed without additional purification. 0.1 M PBS solution was prepared using ultrapure deionized (DI) water from the Millipore Milli-Q system, boasting an electrical resistivity of 18 MΩ.

#### AFM Measurements

An FMV-A cantilever (with a length of 225 μm, width of 30 μm, and thickness of 2.75 μm) surface served as the deposition site for both *E. coli* bacteria and uric acid. The Asylum Research Oxford Jupiter AFM system was employed to evaluate cantilever characteristics under ambient conditions. Thermal noise data extracted from the cantilevers facilitated the identification of individual eigenmodes, enabling subsequent determination of frequency shifts and measurement of spring constants for both pristine and modified cantilevers. In our experimental procedure, we initially coated the cantilever with a PBS solution and conducted measurements of the thermal noise spectra. This enabled us to determine the resonance frequency and spring constant, establishing a baseline for our study. Subsequently, we applied a coating of PBS solution containing *E. coli* and uric acid at varying concentrations onto the cantilever. By analyzing the thermal noise spectra, we measured the modified constant and resonance frequency. The first eigenfrequency of the cantilever was measured to be 66 kHz, and the corresponding spring constant was measured to be 2.48 N/m. For the modified cantilevers, the concentrations of *E. coli* bacteria and uric acid molecules were varied.

## Supporting Information Available

Analytical calculation for the critical frequency and the critical height as a consequence of the added adsorbed layer on a pristine cantilever; calculation of the mass of the protein layers using eq 1 for different cantilevers; frequency shift and changes in spring constant for the modified FMV-A cantilever modified with DPPC bilayer and DPPC/cholesterol assembly; and frequency shift and changes in spring constant for the modified FMV A cantilever modified with *E. coli* bacteria and uric acid molecules (PDF)
