# Correction to “Mass and Stiffness Deconvolution in Nanomechanical Resonators for Precise Mass Measurement and In Vivo Biosensing”

**DOI:** 10.1021/acsnano.5c12144

**Published:** 2025-08-01

**Authors:** Gourav Bhattacharya, Stuart McMichael, Indrianita Lionadi, Pardis Biglarbeigi, Dewar Finlay, Pilar Fernandez-Ibanez, Amir Farokh Payam

In our original article, we would like to report two typographical errors in the equations:

1. Equation 2 in the main text:

This equation should match eq S5 in the Supporting Information. The corrected form is

$$k_{c+a}=\frac{3b_{c}{EI}_{eq}}{L_{c}^{3}}$$

The cantilever width *b*_*c*_ in the numerator was inadvertently omitted due to a typographical error.

2. Equation 3 in the main text and eq S2 in the Supporting Information:

The corrected expression for *EI*_*eq*_ is

$${EI}_{eq}=\frac{E_{c}h_{c}^{3}}{3}+\frac{E_{a}h_{a}^{3}}{3}+\left(h-h_{c}\right)\left(E_{c}h-E_{a}h_{a}\right)h_{c}+\left(h-h_{c}\right)E_{a}h_{a}\left(h-h_{a}\right)$$

Please note that all simulations and experimental results reported in the article were based on the correct equations above. These corrections do not affect the outcomes, analysis, or conclusions of the study. The authors sincerely apologize for these unintended typos.

