# Supplementary material for: Mass and Stiffness Deconvolution in Nanomechanical Resonators for Precise Mass Measurement and In Vivo Biosensing
Source: ACS Nano. 2024 Jul 29;18(31):20181–90. doi: 10.1021/acsnano.4c03391 (PMC11308922; doi:10.1021/acsnano.4c03391)
Supplement: Supplementary file 1 — nn4c03391_si_001.pdf [file nn4c03391_si_001.pdf]

Supplementary Information

Mass and Stiffness Deconvolution in Nanomechanical Resonators for Precise Mass Measurement and In-vivo Biosensing

Gourav Bhattacharya[1], Stuart McMichael[1], Indrianita Lionadi[1], Pardis Biglarbeigi[2], Dewar Finlay[1], Pilar Fernandez-Ibanez[1], Amir Farokh Payam[1, *]

[1]Nanotechnology and Integrated Bioengineering Centre, School of Engineering, Ulster University, BT15 1AP, Belfast, UK.

[2]Department of Pharmacology & Therapeutics, University of Liverpool, Whelan Building, Liverpool, L69 3GE, England, UK.

We assume that the cantilever is singly clamped at one end and free at the other end. The length of the cantilever is L_c, width b_c and thickness h_c oriented with the x-axis with flexural displacement along the z-axis. The adsorbed layer has the same length and width as the cantilever and its thickness is h_a.

By neglecting rotatory inertia and shear deformation, the flexural displacement of the cantilever with coated material obeys the following differential equation for unit width:

$$\frac{\partial^2}{\partial x^2}\left(EI_{eff}\frac{\partial^2 u(x,t)}{\partial x^2}\right) + (\rho_c h_c + \rho_a h_a)\frac{\partial^2 u(x,t)}{\partial t^2} = 0 \quad \text{(S1)}$$

Where EI_{eff} is described as[1,2]:

$$EI_{eff} = \frac{E_c h_c^3}{3} + \frac{E_a h_a^3}{3} + h(E_a h - E_c h_c)\left(E_c h_c^2 - E_a h_a^2\right) + E_a h_a\left(\left(E_c h_c^2 - E_a h_a^2\right)^2 - 2h^2\right) \quad \text{(S2)}$$

Where h is the position of the neutral axis because of the added top layer thickness:

$$h = \frac{E_c h_c^2 + E_a\left(h_a^2 + 2h_a h_c\right)}{2(E_a h_a + E_c h_c)} \quad \text{(S3)}$$

The density of the cantilever and added layer are denoted by ρ_c and ρ_a, respectively.

Solving equation (S1) by adding equation (S2) in (S1) leads to the following equation for the frequency of the cantilever:

$$f + \Delta f = \frac{1}{2\pi}\sqrt{\frac{k_{c+A}}{M_{c+A}}} \quad \text{(S4)}$$

Where

$$k_{c+A} = \frac{3b_c EI_{eff}}{L_c^3} \quad \text{(S5)}$$

$$M_{c+A} = 0.24 L_c b_c(\rho_c h_c + \rho_a h_a) \quad \text{(S6)}$$

While for the pristine cantilever the frequency is calculated by:

$$f = \frac{1}{2\pi}\sqrt{\frac{k_c}{M_c}} \quad \text{(S7)}$$

Where

$$k_c = \frac{b_c E_c h_c^3}{4L_c^3} \quad \text{(S8)}$$

$$M_c = 0.24 L_c b_c \rho_c h_c \quad \text{(S9)}$$

To find the analytical equations for critical height where the polarity of frequency shift changes, we can use the following equality where the frequency of the coated microcantilever (S4) equals the pristine one (S7) which means the frequency

shift would be zero. Then if the right term is higher than the left term the frequency shift would be positive and if the left term is higher than the right term, the frequency shift would be negative:

$$\frac{E_c h_c^2}{4L_c^3 \rho_c} = \frac{3EI_{eff}}{L_c^3(\rho_c h_c + \rho_a h_a)} \tag{S10}$$

Then, we have:

$$h_a\left(E_c h_c^2 \rho_a\right) = 12EI_{eff}\rho_c - E_c h_c^3 \rho_c \tag{S11}$$

Then, at critical frequency, the critical height is computed by:

$$h_a \geq \frac{\rho_c}{\rho_a}\left(\frac{12EI_{eff} - E_c h_c^3}{E_c h_c^2}\right) \tag{S12}$$

The same way can be used to calculate critical Young's modulus and density.

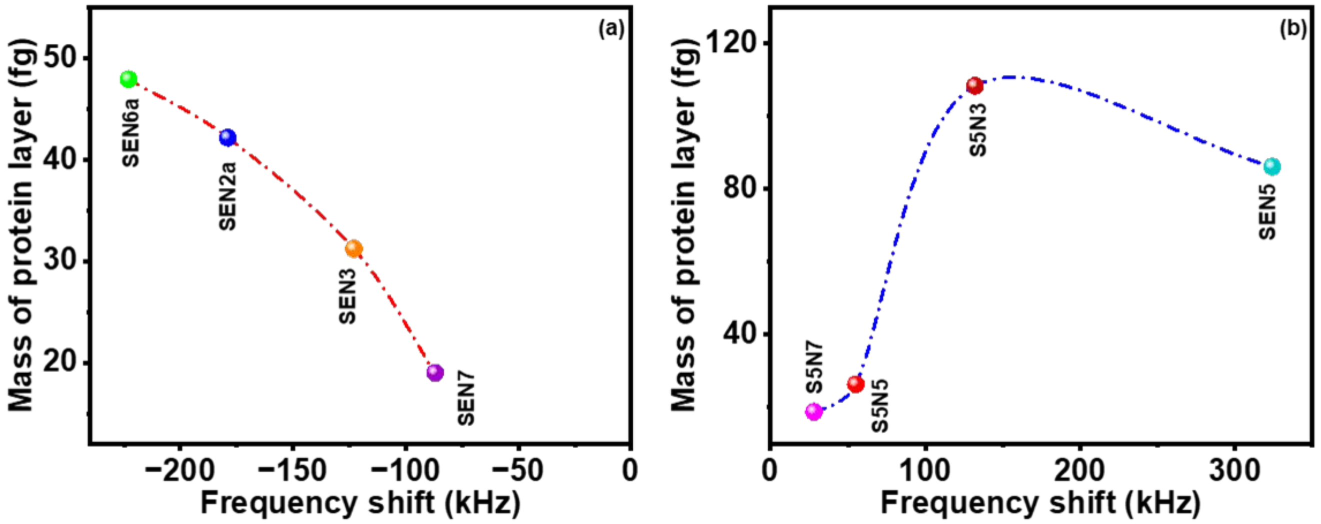

Figure S1 Calculation of mass of the protein layers using equation 1 for different cantilevers (a) from the negative frequency shift and (b) from the positive frequency shift.

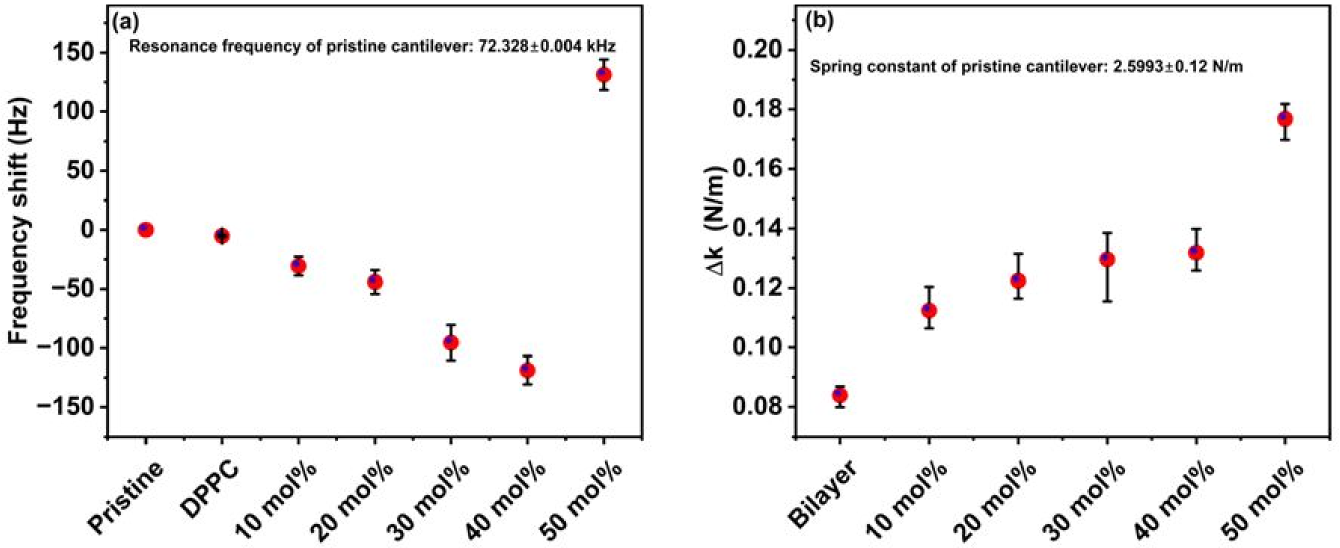

Figure S2 (a) The Frequency shift for the modified FMV-A cantilever modified with DPPC bilayer and DPPC/cholesterol assembly, and (b) changes in spring constant for modified FMV-A cantilever modified with DPPC bilayer and DPPC/cholesterol assembly with different mol% of cholesterol molecules.

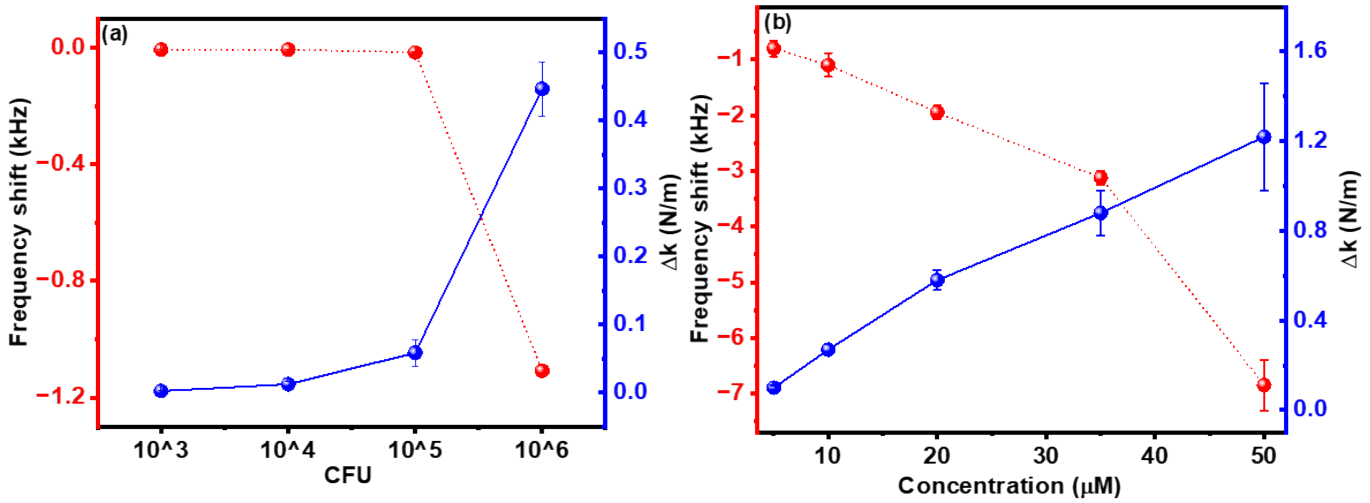

Figure S3 The Frequency shift and changes in spring constant for the modified FMV A cantilever modified with *E. coli* bacteria and Uric acid molecules. (a) Variation frequency shift and spring constant with CFU concentration for *E. coli* bacteria and (b) Variation of frequency shift and spring constant with uric acid concentration. Here we have used a pristine FMV A cantilever with a natural frequency (in air) of 66 kHz and a spring constant of 2.48 N/m.

References

1. Whiting, R., Angadi, M. A. & Tripathi, S. Evaluation of elastic moduli in thin-film/substrate systems by the two-layer vibrating reed method. *Mater. Sci. Eng. B* **1995**, 30, 35–38.
2. Zgheib, E., Alhussein, A., Slim, M. F., Khalil, K. & François, M. Multilayered models for determining the Young's modulus of thin films by means of Impulse Excitation Technique. *Mech. Mater.* **2019**, 137, 103143.
